# Changes in general and COVID-19 vaccine hesitancy among U.S. adults from 2021 to 2022

**DOI:** 10.1080/07853890.2024.2357230

**Published:** 2024-05-30

**Authors:** Kimberly H. Nguyen, E. Lisa Chung, Cheyenne McChesney, Lavanya Vasudevan, Jennifer D. Allen, Robert A. Bednarczyk

**Affiliations:** a Hubert Department of Global Health, Emory University Rollins School of Public Health, Atlanta, GA, USA; b Department of Epidemiology, Emory University Rollins School of Public Health, Atlanta, GA, USA; c Department of Public Health and Community Medicine, Tufts University School of Medicine, Boston, MA, USA; d Department of Community Health, Tufts University, Medford, MA, USA; e Emory Vaccine Center, Emory University, Atlanta, GA, USA

**Keywords:** Vaccine hesitancy, vaccine confidence, COVID-19, routinely recommended vaccines, changes, disparities, adults, nationally representative survey, United States

## Abstract

**Introduction:**

Understanding changes in vaccine hesitancy, overall and by sociodemographic characteristics, may highlight sub-populations for whom more intensive efforts are needed to increase vaccine uptake and confidence.

**Methods:**

We analyzed data using the CDC’s Research and Development Survey (RANDS), a nationally representative survey of U.S. adults ≥18 years, collected from 17 May 2021–30 June 2021 (*n* = 5,458) and 3 November 2022–12 December 2022 (*n* = 6,821). We assessed changes in vaccine hesitancy, changes in vaccine attitudes and attitudes, and factors associated with hesitancy toward both vaccines in general and COVID-19 vaccines among a nationally representative sample of U.S. adults.

**Results:**

Although COVID-19 vaccination (≥1 dose) increased from 67.2% (2021) to 74.7% (2022), COVID-19 vaccine hesitancy increased from 40.7% to 44.6% during the same period. During the same period, hesitancy toward both COVID-19 vaccines and vaccines in general increased among those who were aged ≥65 years and who were non-Hispanic White. However, COVID-19 vaccine hesitancy decreased among non-Hispanic Black adults. Current or former smokers were more hesitant toward vaccines in general (aPR = 1.13, 95%CI: 1.03–1.24) and toward COVID-19 vaccines (aPR = 1.08, 95%CI: 1.01–1.16) compared to never smokers. Among adults who did not receive any COVID-19 vaccines, COVID-19 vaccine hesitancy increased from 86.6% in 2021 to 92.4% in 2022. Furthermore, belief in the overall social benefit of the COVID-19 vaccine decreased from 47.5% to 25.1%.

**Conclusion:**

This study highlights concerning trends in vaccine hesitancy and uptake of the COVID-19 and other recommended vaccines. We found that some high-risk groups (e.g. smokers) and population subgroups have become more vaccine hesitant, suggesting the need for improved and intensified strategies to increase vaccine confidence and uptake. Future research may focus on qualitative inquiry to understand specific concerns and determinants contributing to increased hesitancy among these groups to help inform interventions and communication campaigns to support vaccination.

## KEY MESSAGES

- COVID-19 vaccine hesitancy increased from 40.7% in 2021 to 44.6% in 2022.
- During the same period, hesitancy regarding both the COVID-19 vaccine and vaccines in general increased among those who were ≥65 years and non-Hispanic White while hesitancy toward COVID-19 vaccines decreased among non-Hispanic Black adults.
- Belief in the overall social benefit of the COVID-19 vaccine decreased from 47.5% to 25.1%, sug­gesting need to frame messaging on benefits such as protection from severe disease outcomes.

## Introduction

Vaccine hesitancy, defined as the delay in acceptance or refusal of vaccines despite availability of vaccination services, is a barrier to vaccination and threatens to impede the progress made in preventing vaccine preventable diseases [1]. Despite vaccination being one of the most significant public health achievements, the rising prevalence of vaccine hesitancy worldwide over the past few decades has led the World Health Organization (WHO) in 2019 to declare it as one of the top 10 threats to global health [2]. Prior to the COVID-19 pandemic, there have been outbreaks of vaccine preventable diseases, such as measles, which saw a 30% increase in cases globally in 2019 [3–5]. HPV vaccination coverage in the United States also continued to lag behind other routinely recommend vaccines, with vaccine hesitancy as a significant barrier [6,7]. Approval of COVID-19 vaccines brought a new wave of vaccine hesitancy in the United States, with main reasons for non-vaccination being concerns about efficacy and side effects, lack of trust in vaccines or the government, and the belief that the vaccine is not needed [8–11]. Despite recommendations that everyone 6 months and older receive COVID-19 booster vaccines, coverage is low with only 20.5% of adults having received the updated booster dose as of May 2023, and disparities continue to persist by age, sex, race/ethnicity, income status, geographic region, and other socioeconomic factors [12–16].

Several studies have examined changes in hesitancy toward routinely recommended vaccines and COVID-19 vaccines during the course of the pandemic. For example, studies found increased hesitancy and concerns about the safety and side effects of routine childhood vaccines and lower vaccination coverage during the COVID-19 pandemic [17–20]. Other studies found changes in intentions and attitudes toward the COVID-19 vaccine during the pandemic, such as an increase in concerns about safety and side effects [21,22]. Understanding changes in routinely recommended and COVID-19 specific vaccine hesitancy during the pandemic, overall and by sociodemographic characteristics, may help in the identification of specific sub-populations in need of supportive strategies to increase vaccine uptake and confidence – now and in the future. This study examined changes in vaccine hesitancy, changes in vaccine attitudes and attitudes, and factors associated with hesitancy toward both routinely recommended adult vaccines and COVID-19 vaccines among a nationally representative sample of U.S. adults.

## Methods

### Study design

The Centers for Disease Control and Prevention (CDC)’s Research and Development Survey (RANDS) is a series of probability-sampled, nationally representative, commercial panel surveys of U.S. adults ≥18 years [23]. The methodology of this survey has been published previously [24]. NCHS added questions to the RANDS during COVID Round 3 (2021) and RANDS 7 (2022) to include COVID-19 vaccination coverage, hesitancy, and related health outcomes. The surveys were fielded in a dual mode (web and telephone interviews) from 17 May 2021–30 June 2021 and from 3 November 2022–December 12, 2022. Participants were informed about the purpose of the survey and participation in the web and telephone interviews implied consent. The RANDS during COVID Round 3 (2021) survey had a sample size and response rate of 5,458 (69.5%) and the RANDS 7 (2022) survey had a sample size and response rate of 6,821 (66.1%) [23]. Data were weighted to account for sample design and further weighted to U.S. population counts to account for nonresponse and noncoverage bias [23]. Per Emory University Institutional Review Board determination assessments, this study is not considered human subjects research.

### General vaccine hesitancy and attitudes

Questions on vaccine hesitancy in the RANDS surveys were based on previously validated questions [25]. Hesitancy toward vaccines in general was assessed by the following question: “Overall, how hesitant about vaccines in general would you consider yourself to be?” Response options of “Not at all hesitant” and “Not that hesitant” were combined into a “Not hesitant” category, and responses of “Somewhat hesitant” and “Very hesitant” were combined into a “Hesitant” category. Other questions referred to general vaccines, in which response options were yes/no, and included: 1) concerns about serious, long-term side effects that impacted your decision to get vaccinated, 2) knowledge of anyone who has had a serious, long-term side effect from a vaccine, 3) belief that doctor or health provider is the most trusted source of information about vaccines, 4) confidence that the benefits of vaccines outweigh their risks, and 5) belief that getting vaccinated helps protect others from getting disease.

### COVID-19 vaccine hesitancy, coverage, and attitudes

COVID-19 vaccine hesitancy was assessed by the following question: “Thinking specifically about potential COVID-19 vaccines, how hesitant would you consider yourself to be?” Response options and re-categorization were identical to the general vaccine hesitancy question. To assess COVID-19 vaccination, respondents were asked: “Have you already received a COVID-19 vaccine” (2021) or “Have you had at least one dose of a COVID-19 vaccination” (2022). To assess attitudes toward the COVID-19 vaccines, respondents were asked: “When thinking about [receiving/your plan to get/your plan not to get/whether to get] a COVID-19 vaccine, which of the following, if any, were you thinking about” (2021) or “When answering the previous question about your hesitance towards the COVID-19 vaccines, which of the following, if any, were you thinking about” (2022). Response options were: 1) Overall social benefit of vaccine, 2) long-term health impact, 3) speed of development, 4) government approval process, 5) personal risk of getting vaccinated, 6) risk of contracting COVID-19, 7) information you received from a medical provider, 8) information you received from friends or social media, 9) previous experiences with vaccines, or 10) something else.

### Sociodemographic variables

Sociodemographic variables describing the respondents included: age group (18–29, 30–39, 40–49, 50–64, and ≥65 years), sex (male, female), race and ethnicity (non-Hispanic (NH) White, NH Black, Hispanic, and NH other or multiple races), highest educational attainment (high school graduate or less, some college, college graduate or above), annual household income (<$35,000, $35,000–$49,999, $50,000–$74,999, ≥$75,000), health insurance status (yes, no), and U.S. Census region (Northeast, Midwest, South, and West). Usual place for healthcare was defined as having a usual place “to go to if you are sick and need health care.”

### Health-related variables

High risk conditions were defined according to CDC’s list of conditions that increase risk for severe COVID-19, such as ever having hypertension, coronary heart disease, asthma, chronic obstructive pulmonary disease, emphysema, chronic bronchitis, cancer, heart attack, diabetes, Alzheimer’s disease, dementia, or other cognitive impairment disorder [26]. Anxiety or depression was determined by previously validated two-item Patient Health Questionnaire (PHQ-2) and a two-item Generalized Anxiety Disorder (GAD-2) scale in 2021. The questions from the PHQ-2 were: 1) “Over the last 2 weeks, how often have you been bothered by … having little interest or pleasure in doing things? Would you say not at all, several days, more than half the days, or nearly every day?” 2) “Over the last 2 weeks, how often have you been bothered by … feeling down, depressed, or hopeless? Would you say not at all, several days, more than half the days, or nearly every day?” Questions from the GAD-2 were: “Over the last 2 weeks, how often have you been bothered by the following problems … Feeling nervous, anxious, or on edge? Would you say not at all, several days, more than half the days, or nearly every day?” 2) “Over the last 2 weeks, how often have you been bothered by the following problems … Not being able to stop or control worrying? Would you say not at all, several days, more than half the days, or nearly every day?” For each scale, responses were assigned a numerical value: not at all = 0, several days = 1, more than half the days = 2, and nearly every day = 3. The two responses for each scale were summed and a score equal to three or greater on the PHQ-2 was categorized as symptoms of depression (hereafter referred to as depression) (9). A sum equal to three or greater on the GAD-2 was categorized as symptoms of anxiety (hereafter referred to as anxiety). Adults who had either symptom of anxiety or depression were categorized as having either disorder [27]. In 2022, anxiety or depression was defined as “ever been told by a doctor or other health professional that you had any type of anxiety disorder” or “…any type of depression.” Smoking status was defined as ever smoking at least 100 cigarettes in lifetime. Those who responded “yes” were categorized as a “current or former smoker.” Those who were not current or former smokers were also referred to as “never smokers.”

### Statistical analysis

Hesitancy toward COVID-19-specific vaccines and vaccines in general were assessed overall and by sociodemographic characteristics by year. Differences in the prevalence of general and COVID-19 specific hesitancy by year, and 95% confidence intervals for the differences, were assessed overall and by sociodemographic characteristic. Furthermore, differences in four mutually exclusive groups of general and COVID-19 specific vaccine hesitancy were assessed between 2021 and 2022: 1) hesitant toward vaccines in general only, 2) hesitant toward COVID-19 vaccines only, 3) hesitant toward vaccines in general and COVID-19 vaccines, and 4) neither hesitant toward vaccines in general nor COVID-19 vaccines. Prevalence ratios from multivariable logistic regression via predictive marginals was conducted to assess factors associated with general- and COVID-19-specific vaccine hesitancy in 2021 and 2022 (age, sex, race/ethnicity, highest educational attainment, annual household income, insurance status, region, high-risk conditions, anxiety or depression, smoking status, usual place of healthcare, COVID-19 vaccination status, COVID-19 vaccine hesitancy, hesitancy toward vaccines in general) [28–30]. Furthermore, attitudes toward routinely recommended vaccines were assessed by hesitancy status. Factors that respondents thought of when considering a COVID-19 vaccine were assessed by four mutually exclusive groups of vaccination and hesitancy status: 1) vaccinated/not hesitant, 2) vaccinated/hesitant, 3) not vaccinated/hesitant, 4) not vaccinated/not hesitant. Analyses were weighted to population totals and adjusted for households having multiple telephone lines, unit non-response, and non-coverage of non-cellular-telephone households [23]. Weighted estimates, along with 95% confidence intervals (CIs), were calculated using SAS and Stata 18 to account for the complex survey design. Only significant estimates are presented in the text of this article.

## Results

Sociodemographic characteristics among adults in 2021 (*n* = 5,458) were similar to those in 2022 (*n* = 6,821) (Table 1). For example, the most common characteristics across both survey years were adults who were 50-64 years (24.7%; 24.7%), non-Hispanic (NH) White (62.4%; 62.1%), had a high school education or less (37.9%; 38.6%), had annual household income of ≥75,000 (44.4%; 46.2%), had health insurance (91.1%; 89.9%), had a usual place for healthcare (89.7%; 88.4%), and lived in the South (38.2%; 38.3%). In 2021 and 2022, 52.4 to 53.0% of adults had a history of high-risk conditions, 24.1 to 31.7% had anxiety or depression, and 41.8 to 39.6% were current or former smokers.

**Table 1.** Sociodemographic characteristics of U.S. adults, RANDS, 2021 and 2022.

Variables	2021 (*n* = 5,458)	2022 (*n* = 6,821)
	% (95% CI)	% (95% CI)
Age Groups (in years)		
18–29	20.2 (17.9, 22.5)	19.9 (18.4, 21.3)
30–39	17.4 (16.1, 18.8)	17.5 (16.2, 18.8)
40–49	15.7 (14.3, 17.1)	15.9 (14.5, 17.4)
50–64	24.7 (23.0, 26.4)	24.7 (23.2, 26.1)
≥65	22.0 (20.4, 23.6)	22.1 (20.4, 23.7)
Sex		
Male	48.2 (46.5, 50.0)	48.7 (46.9, 50.5)
Female	51.8 (50.0, 53.5)	51.3 (49.5, 53.1)
Race/ethnicity		
Non-Hispanic White	62.4 (59.4, 65.4)	62.1 (58.8, 65.4)
Non-Hispanic Black	12.0 (9.9, 14.1)	12.1 (10.2, 13.9)
Non-Hispanic Other/Multiple Races	16.9 (15.0, 18.8)	17.1 (15.3, 19.0)
Hispanic	8.7 (7.4, 10.0)	8.7 (7.5, 10.0)
Educational Status		
High school or less	37.9 (35.5, 40.3)	38.6 (36.6, 40.6)
Some college or associate degree	27.0 (25.2, 28.7)	26.5 (25.2, 27.8)
Bachelor degree or higher	35.1 (33.1, 37.2)	34.9 (33.1, 36.7)
Annual Household Income		
<$35,000	30.1 (27.9, 32.2)	27.3 (25.3, 29.3)
$35,000–$49,999	11.4 (9.9, 12.9)	10.5 (9.4, 11.5)
$50,000–$74,999	14.1 (12.7, 15.5)	16.0 (14.7, 17.3)
≥ $75,000	44.4 (41.9, 47.0)	46.2 (44.2, 48.3)
Insurance status		
Insured	91.1 (89.7, 92.5)	89.9 (88.6, 91.2)
Not insured	8.9 (7.5, 10.3)	10.1 (8.8, 11.4)
Usual place of healthcare		
Yes	89.7 (88.6, 90.8)	88.4 (87.3, 89.5)
No	10.3 (9.2, 11.4)	11.6 (10.5, 12.7)
Region		
South	38.2 (33.0, 43.5)	38.3 (33.3, 43.4)
Northeast	17.3 (14.6, 19.9)	17.3 (14.6, 19.9)
West	23.9 (19.3, 28.4)	23.7 (20.7, 26.8)
Midwest	20.7 (15.1, 26.2)	20.7 (15.4, 25.9)
High-risk conditions^a^		
Yes	53.0 (51.4, 54.7)	52.4 (50.7, 54.1)
No	47.0 (45.3, 48.6)	47.6 (45.9, 49.3)
Anxiety or depression^b^		
Yes	24.1 (22.8, 25.4)	31.7 (29.8, 33.5)
No	75.9 (74.6, 77.2)	68.3 (66.5, 70.2)
Current or former smoker^c^		
Yes	41.8 (39.9, 43.7)	39.6 (38.0, 41.2)
No	58.2 (56.3, 60.1)	60.4 (58.8, 62.0)
COVID-19 vaccination		
Yes	67.2 (64.8, 69.7)	74.7 (72.9, 76.5)
No	32.8 (30.3, 35.2)	25.3 (23.5, 27.1)

^a^High-risk conditions were defined as having at least one of the following conditions: history of hypertension, coronary heart disease, asthma, chronic obstructive pulmonary disease, emphysema, chronic bronchitis, cancer, heart attack, or diabetes.

^b^In 2021, presence of anxiety or depression was determined by GAD and PHQ scores greater than 3. In 2022, presence of anxiety or depression was determined by the responses to the following questions: 1) “Have you ever been told by a doctor or other health professional that you had any type of anxiety disorder?” and 2) “Have you ever been told by a doctor or other health professional that you had any type of depression?”.

^c^Smoking status was defined as ever smoking at least 100 cigarettes in lifetime. Those who responded “yes” were categorized as a “current or former smoker”. Those who were not current or former smokers were also referred to as “never smokers.”

**Figure 1.**
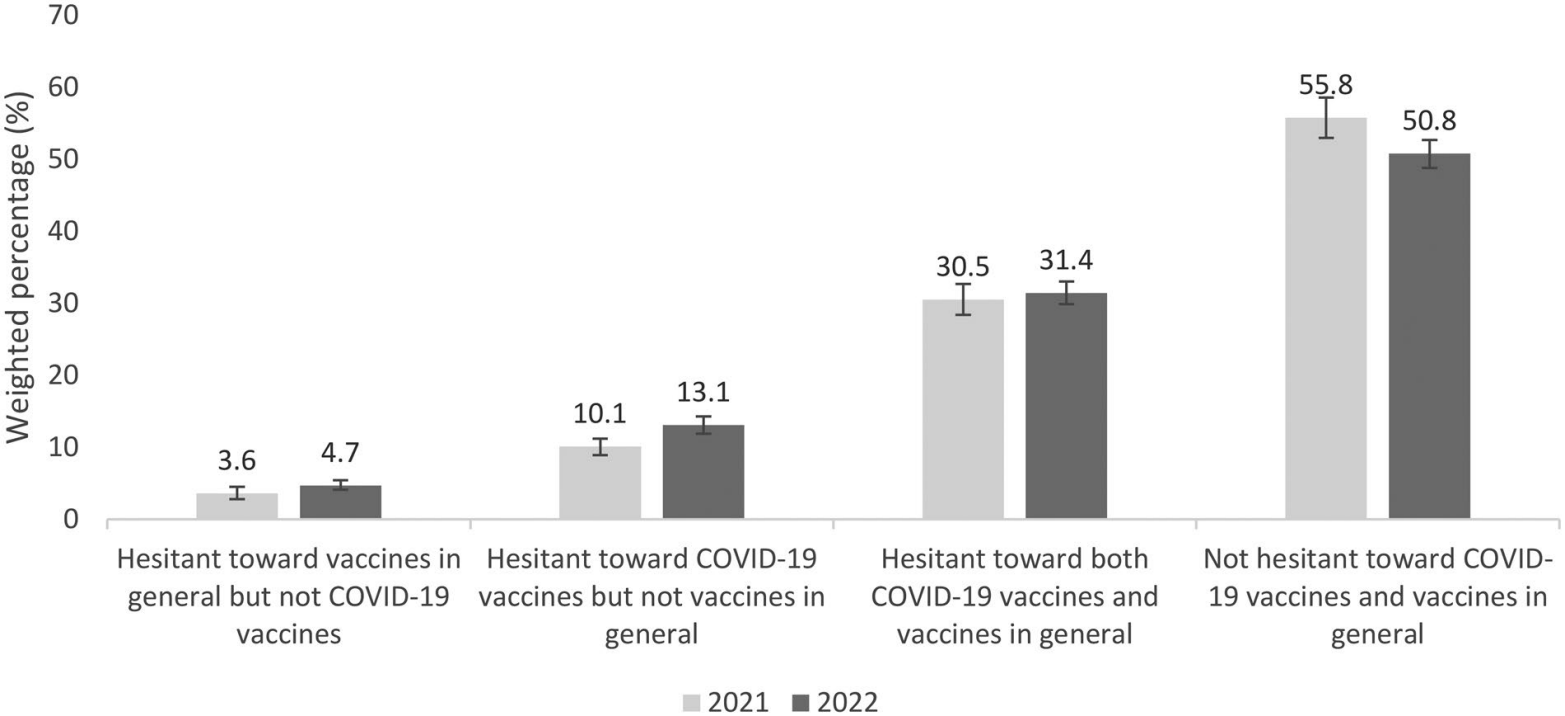
Prevalence of four mutually exclusive vaccine hesitancy groups, RANDS, 2021 and 2022.

Among four mutually exclusive groups in 2022, the proportion of adults who were not hesitant toward COVID-19 vaccines or vaccines in general was highest (50.8%), followed by adults who were hesitant toward both COVID-19 vaccines and vaccines in general (31.4%), adults who were hesitant toward COVID-19 vaccines only (13.1%), and adults who were hesitant toward vaccines in general only (4.7%) (Figure 1). The proportion of adults who were not hesitant about either vaccine decreased from 55.8% to 50.8% (2021 to 2022, respectively), while the proportion of adults who were hesitant about COVID-19 vaccines only increased from 10.1% to 13.1% (2021 to 2022, respectively).

Among non-mutually exclusive groups, there was an increase in hesitancy toward COVID-19 vaccines from 40.7 to 44.6% (Table 2). COVID-19 vaccine hesitancy also increased within 15 of 36 sub-populations examined (adults who were ≥65 years (25.5%; 30.2%), males (35.0%; 41.9%), NH White (38.2%; 43.4%) and NH other/multiple races (45.7%; 54.1%), had a high school education or less (49.9%; 55.8%), had ≥$75,000 annual household income (35.3%; 39.6%), were not insured (52.8%; 63.6%), and had a usual place for healthcare (39.0%; 42.9%). Furthermore, COVID-19 vaccine hesitancy increased among adults who had a high-risk medical condition (31.2%; 42.2%), did not have anxiety or depression (38.9%; 44.2%), and were never smokers (39.4; 43.5%). There was also an increase in COVID-19 vaccine hesitancy among adults who were vaccinated (18.2%; 29.7%) and were not vaccinated against COVID-19 (86.6%; 92.4%), and those who were not hesitant toward vaccines in general (15.3%; 20.5%). Adults who were NH Black (54.7%; 47.8%) had a decrease in COVID-19 vaccine hesitancy from 2021 to 2022. Hesitancy toward vaccines in general also increased from 23.9% to 28.9% among adults ≥65 years, from 31.5% to 34.8% among NH White adults, from 33.6% to 38.8% among adults living in the Midwest from 2021 to 2022, and from 16.3% to 23.5% among those who received a COVID-19 vaccination.

**Table 2.** Prevalence of hesitancy of vaccines in general and COVID-19 vaccines among U.S. adults, RANDS, 2021 and 2022.

	Hesitancy toward vaccines in general			COVID-19 Vaccine hesitancy		
	2021	2022	PD^a^	2021	2022	PD^a^
Variables	% (95% CI)	% (95% CI)	% (95% CI)	% (95% CI)	% (95% CI)	% (95% CI)
Overall	34.6 (32.1, 37.0)	36.4 (34.8, 37.9)	1.8 (−1.1, 4.7)	40.7 (38.1, 43.2)	44.6 (42.6, 46.6)	**4.0 (0.7, 7.2)**
Age Groups (in years)						
18–29	38.4 (32.7, 44.0)	38.0 (33.5, 42.5)	−0.4 (−7.6, 6.8)	47.7 (41.5, 53.9)	52.4 (48.5, 56.3)	4.7 (−2.6, 12.1)
30–39	38.6 (34.9, 42.3)	38.5 (34.6, 42.4)	−0.1 (−5.4, 5.3)	49.4 (45.0, 53.8)	53.3 (49.6, 56.9)	3.9 (−1.8, 9.6)
40–49	37.1 (32.2, 41.9)	38.9 (35.0, 42.9)	1.9 (−4.4, 8.1)	44.1 (38.4, 49.7)	47.4 (42.6, 52.2)	3.4 (−4.0, 10.8)
50–64	36.5 (33.1, 39.9)	38.6 (35.7, 41.5)	2.1 (−2.4, 6.6)	40.4 (37.2, 43.6)	44.2 (40.7, 47.6)	3.8 (−0.9, 8.5)
≥65	23.9 (21.5, 26.3)	28.9 (26.1, 31.7)	**5.0 (1.3, 8.7)**	25.5 (22.9, 28.1)	30.2 (27.0, 33.3)	**4.7 (0.5, 8.8)**
Sex						
Male	29.3 (26.4, 32.3)	32.7 (30.5, 34.9)	3.4 (−0.3, 7.0)	35.0 (31.9, 38.1)	41.9 (40.0, 43.8)	**6.9 (3.2, 10.5)**
Female	39.4 (36.1, 42.8)	39.9 (37.6, 42.1)	0.4 (−3.6, 4.5)	46.1 (42.4, 49.8)	47.3 (44.4, 50.2)	1.2 (−3.5, 5.9)
Race/ethnicity						
Non-Hispanic White	31.5 (28.8, 34.1)	34.8 (32.9, 36.7)	**3.4 (0.1, 6.6)**	38.2 (35.3, 41.1)	43.4 (40.8, 46.0)	**5.2 (1.4, 9.1)**
Non-Hispanic Black	48.7 (45.1, 52.3)	46.2 (41.9, 50.6)	−2.5 (−8.1, 3.2)	54.7 (50.2, 59.3)	47.8 (43.7, 51.8)	**−7.0 (**−**13.1, −0.9)**
Non-Hispanic other/multiple races	39.7 (34.2, 45.1)	39.4 (36.0, 42.8)	−0.3 (−6.7, 6.2)	45.7 (39.9, 51.5)	54.1 (49.1, 59.1)	**8.4 (0.8, 16.1**)
Hispanic	27.3 (22.4, 32.2)	27.9 (22.6, 33.1)	0.5 (−6.6, 7.7)	30.3 (25.0, 35.7)	31.1 (23.8, 38.5)	0.8 (−8.3, 9.9)
Educational Status						
High school or less	42.8 (38.9, 46.8)	46.0 (43.0, 49.1)	3.2 (−1.8, 8.1)	49.9 (45.7, 54.1)	55.8 (52.7, 58.8)	**5.9 (0.7, 11.1)**
Some college	37.3 (34.7, 39.8)	37.2 (35.2, 39.1)	−0.1 (−3.3, 3.1)	44.7 (42.1, 47.3)	46.6 (44.4, 48.9)	2.0 (−1.5, 5.4)
Bachelor degree or higher	23.6 (20.0, 27.2)	25.1 (22.6, 27.7)	1.5 (−2.9, 6.0)	28.1 (24.6, 31.4)	31.4 (28.4, 34.5)	3.4 (−1.2, 7.9)
Annual Household Income						
<$35,000	42.8 (39.1, 46.6)	46.0 (43.4, 48.6)	3.2 (−1.4, 7.7)	49.4 (45.1, 53.6)	52.9 (49.7, 56.1)	3.5 (−1.8, 8.9)
$35,000–$49,999	34.8 (28.4, 41.2)	40.1 (35.2, 45.0)	5.3 (−2.8, 13.3)	39.0 (33.1, 44.9)	45.8 (40.4, 51.2)	6.8 (−1.2, 14.8)
$50,000–$74,999	34.5 (29.8, 39.1)	36.2 (32.0, 40.3)	1.7 (−4.5, 7.9)	41.4 (36.4, 46.4)	45.0 (40.7, 49.3)	3.6 (−3.0, 10.2)
≥$75,000	28.9 (26.5, 31.4)	29.9 (27.7, 32.2)	1.0 (−2.4, 4.3)	35.3 (32.7, 37.9)	39.6 (36.9, 42.4)	**4.4 (0.6, 8.1)**
Insurance status						
Insured	33.1 (30.7, 35.5)	34.8 (33.2, 36.5)	1.7 (−1.2, 4.9)	39.5 (36.9, 42.1)	42.8 (40.7, 44.8)	3.28 (−0.03, 6.6)
Not insured	49.3 (40.6, 58.0)	49.8 (45.2, 54.3)	0.5 (−9.3, 10.3)	52.8 (46.6, 59.1)	63.6 (58.7, 68.6)	**10.8 (2.8, 18.8)**
Usual place of healthcare						
Yes	33.4 (30.9, 35.8)	35.2 (33.5, 36.9)	1.8 (−1.1, 4.8)	39.0 (36.4, 41.5)	42.9 (40.7, 45.0)	**3.9 (0.5, 7.2)**
No	45.0 (37.7, 52.2)	45.0 (39.3, 50.7)	0.0 (−9.2, 9.2)	55.9 (48.3, 63.6)	58.6 (54.0, 63.2)	2.7 (−6.3, 11.6)
Region						
South	37.1 (32.8, 41.3)	38.1 (35.8, 40.5)	1.1 (−3.8, 5.9)	42.8 (38.2, 47.5)	48.5 (45.2, 51.8)	5.6 (−0.1, 11.3)
Northeast	35.3 (30.2, 40.4)	35.9 (32.8, 39.0)	0.6 (−5.4, 6.6)	39.1 (33.8, 44.3)	41.6 (38.3, 45.0)	2.6 (−3.7, 8.8)
West	30.8 (26.8, 34.8)	31.7 (29.2, 34.3)	0.9 (−3.9, 5.6)	37.1 (32.9, 41.2)	38.8 (35.1, 42.5)	1.7 (−3.9, 7.3)
Midwest	33.6 (30.3, 36.9)	38.8 (35.4, 42.1)	**5.2 (0.5, 9.9)**	42.3 (37.8, 46.7)	46.7 (42.8, 50.6)	4.5 (−1.4, 10.3)
High-risk conditions^b^						
Yes	33.0 (30.1, 35.9)	34.0 (32.0, 35.9)	1.0 (−2.5, 4.5)	31.2 (35.1, 41.3)	42.2 (39.5, 44.9)	**11.1 (6.9, 15.2)**
No	36.4 (33.3, 39.4)	39.0 (36.6, 41.4)	2.7 (−1.2, 6.6)	43.5 (40.4, 46.6)	47.4 (44.8, 50.0)	3.9 (−0.1, 8.0)
Anxiety or depression^c^						
Yes	37.9 (33.4, 42.4)	36.6 (34.0, 39.2)	−1.3 (−6.5, 3.9)	46.3 (41.3, 51.3)	45.6 (41.7, 49.6)	−0.7 (−7.1, 5.7)
No	33.5 (31.3, 35.7)	36.3 (34.3, 38.2)	2.8 (−0.2, 5.7)	38.9 (36.6, 41.3)	44.2 (42.1, 46.3)	**5.2 (2.1, 8.4)**
Current or former smoker^d^						
Yes	36.8 (33.7, 39.9)	39.3 (37.1, 41.6)	2.6 (−1.3, 6.4)	42.4 (39.0, 45.8)	46.4 (43.7, 49.1)	4.0 (−0.3, 8.4)
No	32.9 (30.2, 35.7)	34.5 (32.4, 36.6)	1.6 (−1.9, 5.0)	39.4 (36.3, 42.5)	43.5 (41.0, 46.1)	**4.1 (0.1, 8.1)**
COVID-19 vaccination						
Yes	16.3 (14.3, 18.2)	23.5 (22.0, 25.0)	**7.2 (4.7, 9.7)**	18.2 (16.1, 20.4)	29.7 (27.9, 31.5)	**11.5 (8.7, 14.3)**
No	70.9 (68.0, 74.0)	74.1 (70.9, 77.4)	3.2 (−1.2, 7.6)	86.6 (83.5, 89.8)	92.4 (90.9, 94.0)	**5.8 (2.3, 9.3)**
COVID-19 vaccine hesitancy						
Yes	75.2 (72.7, 77.6)	70.6 (68.4, 72.7)	**−4.6 (**−**7.9, −1.3)**	–	–	–
No	6.1 (4.7, 7.6)	8.5 (7.3, 9.6)	**2.4 (0.5, 4.3)**	–	–	–
Hesitant towards vaccines in general						
Yes	–	–	–	89.3 (87.2, 91.5)	87.0 (85.2. 88.7)	−2.3 (−5.1, 0.5)
No	–	–	–	15.3 (13.4, 17.2)	20.5 (18.6, 22.5)	**5.2 (2.5, 7.9)**

Note: Boldface denotes statistical significance (*p* < .05).

^a^Prevalence difference.

^b^High-risk conditions were defined as having at least one of the following conditions: history of hypertension, coronary heart disease, asthma, chronic obstructive pulmonary disease, emphysema, chronic bronchitis, cancer, heart attack, or diabetes.

^c^In 2021, presence of anxiety or depression was determined by GAD7 and PHQ scores greater than 3. In 2022, presence of anxiety or depression was determined by the responses to the following questions: 1) “Have you ever been told by a doctor or other health professional that you had any type of anxiety disorder?” and 2) “Have you ever been told by a doctor or other health professional that you had any type of depression?”.

^d^Smoking status was defined as ever smoking at least 100 cigarettes in lifetime. Those who responded “yes” were categorized as a “current or former smoker”. Those who were not current or former smokers were also referred to as “never smokers.”.

Factors associated with hesitancy toward vaccines in general and COVID-19 vaccines differed between 2021 and 2022 (Table 3). In general, older adults (≥65 years) and those with higher education (some college or Bachelor’s degree or higher) were less likely to be hesitant toward vaccines in general or COVID-19 vaccines compared to their respective counterparts. However, those who were females and NH Black were more like to be hesitant toward vaccines in general and COVID-19 vaccines compared to their respective counterparts. In 2022, adults 30–39 years (aPR = 1.14, 95%CI: 1.05, 1.25) and current or former smokers were more hesitant toward COVID-19 vaccines (aPR = 1.08, 95%CI: 1.01–1.16) compared to their respective counterparts.

**Table 3.** Factors associated with hesitancy toward vaccines in general and COVID-19 vaccines, RANDS, 2021 and 2022.

	2021		2022	
	Hesitant toward vaccines in general	Hesitant toward COVID-19 vaccines	Hesitant toward vaccines in general	Hesitant toward COVID-19 vaccines
Variables	aPR^a^ (95% CI)	aPR^a^ (95% CI)	aPR^a^ (95% CI)	aPR^a^ (95% CI)
Age Groups (in years)				
18–29 (Reference)	1.00	1.00	1.00	1.00
30–39	1.13 (0.95, 1.36)	1.14 (0.98, 1.33)	1.12 (0.97, 1.29)	**1.14 (1.05, 1.25)**
40–49	1.10 (0.90, 1.34)	1.01 (0.87, 1.18)	1.20 (1.03, 1.39)	1.04 (0.94, 1.15)
50–64	1.00 (0.85, 1.17)	0.87 (0.76, 1.00)	1.10 (0.95, 1.28)	0.90 (0.81, 1.00)
≥65	**0.67 (0.56, 0.80)**	**0.55 (0.47, 0.63)**	**0.84 (0.73, 0.97)**	**0.62 (0.56, 0.69)**
Sex				
Male (Reference)	1.00	1.00	1.00	1.00
Female	**1.31 (1.17, 1.47)**	**1.28 (1.15, 1.43)**	**1.18 (1.07, 1.30)**	**1.13 (1.05, 1.21)**
Race/ethnicity				
Non-Hispanic White (Reference)	1.00	1.00	1.00	1.00
Non-Hispanic Black	**1.25 (1.09, 1.43)**	**1.18 (1.04, 1.33)**	**1.15 (1.03, 1.28)**	0.95 (0.84, 1.07)
Non-Hispanic other/multiple races	1.09 (0.95, 1.25)	1.01 (0.90, 1.14)	1.01 (0.88, 1.14)	1.08 (0.97, 1.20)
Hispanic	0.88 (0.71, 1.10)	**0.77 (0.62, 0.97)**	0.86 (0.71, 1.05)	**0.76 (0.60, 0.95)**
Educational Status				
High school or less (Reference)	1.00	1.00	1.00	1.00
Some college	**0.89 (0.80, 0.99)**	0.91 (0.82, 1.00)	**0.86 (0.78, 0.95)**	**0.85 (0.78, 0.92)**
Bachelor degree or higher	**0.61 (0.52, 0.71)**	**0.60 (0.52, 0.68)**	**0.62 (0.54, 0.70)**	**0.59 (0.54, 0.65)**
Annual Household Income				
<$35,000 (Reference)	1.00	1.00	1.00	1.00
$35,000–$49,999	0.95 (0.78, 1.15)	0.92 (0.79, 1.08)	0.94 (0.82, 1.07)	0.96 (0.84, 1.10)
$50,000–$74,999	0.98 (0.85, 1.12)	0.99 (0.87, 1.13)	0.90 (0.79, 1.02)	0.98 (0.88, 1.09)
≥ $75,000	0.89 (0.79, 1.01)	0.93 (0.84, 1.03)	0.83 (0.74, 0.93)	0.97 (0.89, 1.06)
Insurance status				
Not insured (Reference)	1.00	1.00	1.00	1.00
Insured	0.86 (0.72, 1.03)	0.99 (0.86, 1.15)	0.88 (0.78, 1.00)	**0.87 (0.80, 0.95)**
Region				
South (Reference)	1.00	1.00	1.00	1.00
Northeast	1.08 (0.90, 1.29)	1.04 (0.89, 1.21)	1.04 (0.92, 1.17)	0.95 (0.84, 1.07)
West	0.91 (0.78, 1.06)	0.94 (0.81, 1.10)	0.93 (0.82, 1.04)	0.85 (0.75, 0.95)
Midwest	0.99 (0.87, 1.13)	1.04 (0.91, 1.19)	1.06 (0.94, 1.19)	0.99 (0.88, 1.10)
High-risk conditions^b^				
No (Reference)	1.00	1.00	1.00	1.00
Yes	0.98 (0.88, 1.08)	1.00 (0.92, 1.10)	**0.84 (0.78, 0.91)**	0.95 (0.87, 1.02)
Anxiety or depression^c^				
No (Reference)	1.00	1.00	1.00	1.00
Yes	0.93 (0.83, 1.05)	0.98 (0.90, 1.07)	0.93 (0.84, 1.02)	0.94 (0.86, 1.03)
Current or former smoker^d^				
No (Reference)	1.00	1.00	1.00	1.00
Yes	1.09 (0.99, 1.20)	1.06 (0.96, 1.17)	**1.13 (1.03, 1.24)**	**1.08 (1.01, 1.16)**

Note: Boldface denotes statistical significance (*p* < .05).

^a^Adjusted prevalence ratio.

^b^High-risk conditions were defined as having at least one of the following conditions: history of hypertension, coronary heart disease, asthma, chronic obstructive pulmonary disease, emphysema, chronic bronchitis, cancer, heart attack, or diabetes.

^c^In 2021, presence of anxiety or depression was determined by GAD7 and PHQ scores greater than 3. In 2022, presence of anxiety or depression was determined by the responses to the following questions: 1) “Have you ever been told by a doctor or other health professional that you had any type of anxiety disorder?” and 2) “Have you ever been told by a doctor or other health professional that you had any type of depression?”.

^d^Smoking status was defined as ever smoking at least 100 cigarettes in lifetime. Those who responded “yes” were categorized as a “current or former smoker”. Those who were not current or former smokers were also referred to as “never smokers.”.

Among adults who were hesitant and not vaccinated (28.0%) in 2022, the most common concern was long-term health impacts (67.4%), personal risk of getting vaccinated (44.1%), and speed of development (43.4%) (Table 4). From 2021 to 2022, thoughts when thinking about hesitancy toward COVID-19 vaccines decreased among adults who were hesitant and not vaccinated for each of the following: 1) overall social benefit of vaccine (18.9 to 14.1%), 2) speed of development (54.2 to 43.4%), 3) personal risk of getting vaccinated (51.3 to 44.1%), and 4) previous experiences with vaccines (20.9 to 14.1%). For vaccines in general, there was an increase in concerns about serious, long-term side effects (61.9 to 67.9%) and personally knowing someone who had a serious, long-term side effect from a vaccine (24.1 to 31.9%) among hesitant adults (Table 5). In addition, there was a decrease in confidence that the benefits of vaccines outweigh their risks (64.1 to 58.1%) and the belief that the vaccine protects others from getting disease (57.7 to 46.8%).

**Table 4.** Attitudes and beliefs toward COVID-19 vaccines, RANDS, 2021 and 2022.

	2021	2022	PD^a^
	% (95% CI)	% (95% CI)	% (95% CI)
**Overall**			
1. Overall social benefit of vaccine	47.5 (45.7, 49.4)	25.1 (23.7, 26.5)	**−22.5 (**−**24.8, −20.1)**
2. Long-term health impacts	52.0 (50.1, 53.9)	46.7 (45.4, 48.1)	**−5.3 (**−**7.7, −3.0)**
3. Speed of development	36.8 (34.7, 38.9)	27.5 (25.9, 29.1)	**−9.3 (**−**11.9, −6.7)**
4. Government approval process	32.8 (30.7, 35.0)	24.5 (23.1, 25.9)	**−8.3 (**−**10.9, −5.7)**
5. Personal risk of getting vaccinated	36.0 (33.8, 38.1)	26.1 (24.7, 27.5)	**−9.9 (**−**12.4, −7.3)**
6. Risk of contracting COVID-19	49.3 (47.2, 51.5)	31.7 (30.2, 33.1)	**−17.7 (**−**20.3, −15.1)**
7. Information you received from a medical provider	28.7 (27.0, 30.3)	17.7 (16.5, 18.9)	**−10.9 (**−**13.0, −8.9)**
8. Information you received from friends or social media	21.1 (19.5, 22.7)	15.1 (14.0, 16.1)	**−6.1 (**−**8.0, −4.1)**
9. Previous experiences with vaccines	26.5 (24.6, 28.3)	18.0 (16.8, 19.3)	**−8.5 (**−**10.7, −6.2)**
10. Something else, please specify:	5.2 (4.2, 6.2)	8.2 (7.3, 9.0)	**3.0 (1.7, 4.2)**
**Hesitant towards COVID-19 vaccine and did not receive COVID-19 vaccine, 28.0% (25.9, 30.2)**			
1. Overall social benefit of vaccine	18.9 (16.2, 21.6)	14.1 (11.7, 16.5)	**−4.8 (**−**8.5, −1.2)**
2. Long-term health impacts	71.7 (68.2, 75.2)	67.4 (64.1, 70.7)	−4.3 (−9.2, 0.5)
3. Speed of development	54.2 (50.7, 57.7)	43.4 (39.6, 47.2)	**−10.8 (**−**16, −5.7)**
4. Government approval process	34.3 (30.4, 38.2)	34.9 (31.2, 38.6)	0.6 (−4.7, 6.0)
5. Personal risk of getting vaccinated	51.3 (46.8, 55.9)	44.1 (41.2, 47.1)	**−7.2 (**−**12.6, −1.8)**
6. Risk of contracting COVID-19	24.6 (22.4, 26.8)	22.4 (19.4, 25.4)	−2.2 (−6.0, 1.5)
7. Information you received from a medical provider	15.9 (13.2, 18.5)	14.5 (11.7, 17.3)	−1.4 (−5.2, 2.5)
8. Information you received from friends or social media	24.3 (21.5, 27.0)	23.0 (19.7, 26.2)	−1.3 (−5.5, 2.9)
9. Previous experiences with vaccines	20.9 (18.0, 23.9)	14.1 (11.6, 16.7)	**−6.8 (**−**10.7, −2.9)**
10. Something else, please specify:	7.4 (5.5, 9.3)	8.1 (6.6, 9.6)	0.7 (−1.7, 3.1)
**Not hesitant towards COVID-19 vaccine and did not receive COVID-19 vaccine, 4.3% (3.2, 5.5)**			
1. Overall social benefit of vaccine	27.7 (19.5, 35.9)	12.9 (4.3, 21.5)	**−14.8 (**−**26.7, −3.0)**
2. Long-term health impacts	40.3 (29.3, 51.3)	30.4 (19.1, 41.6)	−9.9 (−25.7, 5.8)
3. Speed of development	20.3 (12.0, 28.7)	26.4 (14.0, 38.9)	6.1 (−8.9, 21.1)
4. Government approval process	16.9 (8.9, 24.8)	14.1 (6.6, 21.5)	−2.8 (−13.7, 8.1)
5. Personal risk of getting vaccinated	32.9 (21.0, 44.7)	18.3 (8.4, 28.2)	−14.6 (−30.0, 0.9)
6. Risk of contracting COVID-19	35.4 (25.4, 45.3)	26.2 (14.1, 38.3)	−9.2 (−24.9, 6.5)
7. Information you received from a medical provider	13.1 (5.2, 21.0)	12.4 (4.9, 20.0)	−0.7 (−11.6, 10.2)
8. Information you received from friends or social media	17.4 (6.3, 28.4)	19.5 (7.9, 31.2)	2.1 (−14.0, 18.2)
9. Previous experiences with vaccines	6.9 (3.5, 10.2)	14.0 (2.3, 25.8)	7.1 (−5.1, 19.3)
10. Something else, please specify:	8.1 (0.8, 15.5)	10.9 (2.6, 19.2)	2.8 (−8.3, 13.9)
**Hesitant towards COVID-19 vaccine and received COVID-19 vaccine, 12.3% (10.9, 13.8)**			
1. Overall social benefit of vaccine	42.4 (37.5, 47.3)	17.6 (15.2, 20.1)	**−24.8 (**−**30.2, −19.3)**
2. Long-term health impacts	65.8 (60.6, 71.1)	68.9 (65.5, 72.2)	3.1 (−3.2, 9.3)
3. Speed of development	52.0 (46.2, 57.8)	41.7 (38.0, 45.4)	**−10.3 (**−**17.2, −3.4)**
4. Government approval process	34.7 (30.4, 39.0)	30.9 (29.9, 34.8)	−3.8 (−8.8, 1.1)
5. Personal risk of getting vaccinated	50.7 (44.9, 56.4)	35.0 (31.2, 38.8)	**−15.7 (**−**22.6, −8.8)**
6. Risk of contracting COVID-19	49.4 (43.8, 54.9)	24.8 (21.7, 27.9)	**−24.6 (**−**30.8, −18.2)**
7. Information you received from a medical provider	30.8 (26.9, 34.8)	12.0 (9.9, 14.1)	**−18.8 (**−**23.4, −14.4)**
8. Information you received from friends or social media	27.7 (24.0, 31.5)	18.4 (16.0, 20.8)	**−9.3 (**−**13.7, −4.9)**
9. Previous experiences with vaccines	22.3 (17.4, 27.3)	17.1 (14.7, 19.6)	−5.2 (−10.8, 0.3)
10. Something else, please specify:	6.8 (4.9, 8.7)	9.4 (7.9, 11.0)	2.6 (0.2, 5.1)
**Not hesitant towards COVID-19 vaccine and received COVID-19 vaccine, 55.3% (52.5, 58.1)**			
1. Overall social benefit of vaccine	66.5 (64.4, 68.7)	34.0 (31.6, 36.4)	**−32.5 (**−**35.7, −29.3)**
2. Long-term health impacts	40.4 (37.9, 43.0)	31.9 (30.3, 33.5)	**−8.5 (**−**11.5, −5.5)**
3. Speed of development	26.2 (24.0, 28.4)	16.5 (15.1, 17.9)	**−9.7 (**−**12.3, −7.1)**
4. Government approval process	33.8 (31.1, 36.5)	19.3 (17.8, 20.7)	**−14.5 (**−**17.6, −11.4)**
5. Personal risk of getting vaccinated	25.9 (23.6, 28.1)	16.0 (14.3, 17.6)	**−10.0 (**−**12.7, −7.1)**
6. Risk of contracting COVID-19	64.6 (61.9, 67.3)	39.9 (37.8, 42.0)	**−24.7 (**−**28.1, −21.3)**
7. Information you received from a medical provider	36.9 (34.3, 39.5)	22.1 (20.1, 24.1)	**−14.8 (**−**18.1, −11.5)**
8. Information you received from friends or social media	18.6 (16.4, 20.7)	10.6 (9.0, 12.1)	**−8.0 (**−**10.6, −5.4)**
9. Previous experiences with vaccines	32.5 (30.0, 35.1)	20.9 (19.0, 22.7)	**−11.6 (**−**14.7, −8.5)**
10. Something else, please specify:	3.4 (2.7, 4.2)	7.6 (6.5, 8.7)	**4.2 (2.9, 5.6)**

Note: Boldface denotes statistical significance (*p* < 0.05).

^a^Prevalence difference.

**Table 5.** Attitudes and beliefs toward vaccines in general, RANDS, 2021 and 2022.

	2021	2022	PD^a^
	% (95% CI)	% (95% CI)	% (95% CI)
**Overall**			
Is your doctor or health provider your most trusted source of information about vaccines? (Y/N)	51.1 (50.5, 57.7)	52.3 (49.5, 55.0)	1.2 (−3.4, 5.7)
Have you ever had concerns about serious, long-term side effects that impacted your decision to get vaccinated? (Y/N)	28.0 (26.2, 29.9)	36.0 (34.4, 37.5)	**8.0 (5.5, 10.4)**
Do you personally know anyone who has had a serious, long-term side effect from a vaccine? (Y/N)	13.4 (12.3, 14.6)	17.7 (16.2, 19.2)	**4.3 (2.4, 6.1)**
Is your doctor or health provider your most trusted source of information about vaccines? (Y/N)	61.5 (59.5, 63.4)	66.7 (64.7, 68.7)	**5.2 (2.4, 8.0)**
How confident are you that the benefits of vaccines outweigh their risks? (Confident vs Not confident)	85.9 (84.3, 87.5)	82.4 (80.9, 83.9)	**−3.5 (**−**5.7, −1.3)**
Do you believe that getting vaccinated helps protect others from getting disease? (Y/N)	78.8 (76.9, 80.8)	73.0 (71.1, 74.9)	**−5.8 (**−**8.5, −3.1)**
**Hesitant towards vaccines in general**			
Have you ever had concerns about serious, long-term side effects that impacted your decision to get vaccinated? (Y/N)	61.9 (59.0, 64.9)	67.9 (65.6, 70.3)	**6**.**0 (2.2, 9.8)**
Do you personally know anyone who has had a serious, long-term side effect from a vaccine? (Y/N)	24.1 (21.5, 26.6)	31.9 (28.8, 35.1)	**7.8 (3.8, 11.9)**
How confident are you that the benefits of vaccines outweigh their risks? (Confident vs Not confident)	64.1 (60.5, 67.7)	58.1 (55.0, 61.2)	**−6.0 (**−**10.8, −1.2)**
Do you believe that getting vaccinated helps protect others from getting disease? (Y/N)	57.7 (54.1, 61.3)	46.8 (44.0, 49.6)	**−10.9 (**−**15.4, −6.3)**
**Not hesitant towards vaccines in general**			
Have you ever had concerns about serious, long-term side effects that impacted your decision to get vaccinated? (Y/N)	10.2 (9.0, 11.4)	17.8 (16.1, 19.5)	**7.6 (5.5, 9.6)**
Do you personally know anyone who has had a serious, long-term side effect from a vaccine? (Y/N)	7.8 (6.6, 9.0)	9.5 (8.1, 10.9)	1.7 (−0.1, 3.5)
Is your doctor or health provider your most trusted source of information about vaccines? (Y/N)	65.4 (63.2, 67.7)	75.0 (72.4, 77.6)	**9.6 (6.2, 13.0)**
How confident are you that the benefits of vaccines outweigh their risks? (Confident vs. Not confident)	97.5 (96.7, 98.3)	96.2 (95.1, 97.2)	−1.3 (−2.6, 0)
Do you believe that getting vaccinated helps protect others from getting disease? (Y/N)	90.1 (88.2, 92.0)	87.7 (85.9, 89.5)	−2.4 (−5, 0.3)

Note: Boldface denotes statistical significance (*p* < 0.05).

^a^Prevalence difference.

## Discussion

Our study findings show a significant increase in COVID-19 vaccine hesitancy from 2021 to 2022, as well as differences in vaccine hesitancy toward COVID-19 vaccines and vaccines in general among selected sub-populations, including adults >65 years and NH Whites. Given that those aged 65 and older represent 63% of COVID-19 hospitalizations, 61% of all ICU admissions and 90% of all COVID related deaths, these findings are concerning and reflect the need for more intensive efforts to increase vaccine confidence and protect high-risk adults from severe COVID-19 outcomes [31]. Vaccine hesitancy toward COVID-19 and other vaccines increased among adults ≥65 years from 2021 to 2022; however, after adjusting for all study-related variables such as high-risk conditions, adults ≥65 years were not associated with higher hesitancy toward COVID-19 and other vaccines. This suggests that while hesitancy has increased slightly among the older adult population, this group still has the lowest prevalence of hesitancy toward COVID-19 and other vaccines compared to other younger age groups. Furthermore, it is also notable that we observed a decrease in COVID-19 vaccine hesitancy from 2021 to 2022 among some groups, such as NH Black adults. Given efforts to improve vaccine confidence in this population during the pandemic, such as community-based outreach and vaccination strategies, these results suggest that similar efforts could be made to reduce hesitancy and improving vaccination coverage for other subpopulations [32].

COVID-19 vaccine hesitancy also increased among those who reported that they were from “another” or multiple races, had a school education or less, or ever had high risk conditions. Furthermore, being a current or former smoker was also associated with increased hesitancy toward COVID-19 vaccines. These groups represent groups that may be more vulnerable to the effects of COVID-19. While it is critical that targeted messages to advance health equities and protect all people from severe health consequences of COVID-19, there is a particular need to focus on those who are most at risk.

With the increase in hesitancy toward the COVID-19 vaccine, there were also changes in attitudes toward the vaccine. Belief in the overall social benefit of the vaccine decreased from 2021 to 2022 among those who were not vaccinated, which may be reflective of evidence suggesting lower impact of the vaccine on preventing breakthrough COVID-19 infections or the recognition that most people had already been exposed. These results may also reflect concerns about the effectiveness of vaccines, which may have contributed to the increase in COVID-19 vaccine hesitancy and the low coverage of booster doses. These findings also suggest that vaccination programs should emphasize key individual-level benefits of vaccination, including reduced likelihood of severe negative health outcomes from COVID-19 disease. Such individual-level benefits may be conveyed via evidence-based strategies such as provider recommendations for COVID-19 vaccines, in the context of patient-centered conversations to increase patient motivation and decrease barriers such as misinformation and uncertainty. Other approaches that have been tested previously include the engagement of trusted messengers such as community or faith-based leaders to communicate about vaccines [32]. Studies have shown that vaccine uptake can be increased by improving confidence in vaccine safety and efficacy and emphasizing data on lower transmission of COVID-19 from vaccinated individuals to friends and family [33–35]. Tailoring of messages based on each sub-population’s attitudes and norms may help to increase the effectiveness of messages [36].

Among those who were hesitant toward vaccines in general, concerns about serious, long-term side effects, or knowing someone with serious, long-term side effects, increased from 2021 to 2022. These results, along with findings demonstrating that thoughts about long-term health impacts were the main concern among those thinking about getting COVID-19 vaccines, reinforce that risk perception may be a major barrier in vaccine uptake. Furthermore, confidence that the benefits of vaccines outweigh their risks, and the belief that getting vaccinated helps protect others from getting disease, decreased during the same time period. These results suggest that these concerns may be based on perceptions of risk rather than true events and that further efforts are needed to address concerns and misinformation about routinely recommended vaccines, as well as improve confidence in vaccines to protect others from vaccine preventable diseases.

The study also showed that while the proportion of adults who are hesitant toward COVID-19 vaccines increased, the proportion of adults who are neither hesitant toward COVID-19 nor other vaccines has decreased, suggesting that hesitancy may be increasing but only toward COVID-19 vaccines. Understanding reasons for hesitancy toward COVID-19 vaccines, providing targeted messages and strategies to increase vaccination, and recommending COVID-19 vaccines to all eligible individuals are needed to improve uptake and confidence of all vaccines. The U.S. Advisory Committee on Immunization Practices recommends that all individuals aged ≥6 months receive the updated COVID-19 vaccines [37]. To achieve high vaccination coverage, strategies include working with community leaders to promote vaccines, targeted messages to highlight the importance and benefits of vaccination, combating misinformation, offering vaccines at every opportunity, and increasing access to vaccines, particularly in rural or hard to reach areas and among those who are most at risk [32]. The findings from this study can support targeted efforts and messages to increase vaccination uptake and confidence, particularly among populations who are most hesitant.

The main strengths of this study are the generalizability of the findings to the U.S. population and the ability to compare different types of hesitancy across two time periods. These results may also be applicable to other countries with similar socio-demographic characteristics and vaccination landscape. The findings of this study are subject to several limitations. First, although sampling methods and data weighting were designed to produce nationally representative results, the potential for coverage bias is greater in commercial survey panels as compared to traditional household surveys. Second, because responses were self-reported, constructs such as vaccination receipt, hesitancy, and thoughts/attitudes may be subject to recall or social desirability bias. Third, while the general vaccine hesitancy question asks about vaccines in general, it is not clear if respondents were thinking of COVID-19 vaccines. This question was modified from a previously validated question in the National Immunization Survey, which asks “Overall, how hesitant about childhood shots would you consider yourself to be?” [38] While cognitive tests found that parents understood the original question to refer to “childhood vaccines,” it is unknown whether respondents understood the question in this study to refer to routinely recommended vaccines [25]. The original and adapted questions have been fielded in previous data collections of the RANDS and the National Immunization Survey, as well as published in the literature [39, 40]. Fourth, the 2021 survey asks about “potential COVID-19 vaccines,” which may cause misclassification if respondents were confused about the question since the COVID-19 vaccine was already available at the time of data collection. The COVID-19 vaccine hesitancy questions in 2021 were developed, cognitively tested, and approved prior to the availability of vaccines. Once the survey was fielded, the word “potential” was kept for consistency with previously validated questions. In the 2022 survey, the word “potential” was removed from the question, and a similar proportion of respondents answered, “don’t know” (2%), which suggests that the word “potential” did not make a large effect on question comprehension. Finally, the survey did not ask about all possible high-risk conditions or distinguish between current and ever smokers, so results may not be a complete list of all health-related conditions and behaviors.

This study demonstrates the changes in hesitancy toward COVID-19 and other vaccines from 2021 to 2022, factors associated with each type of hesitancy, and changes in attitudes and beliefs toward COVID-19 and other vaccines. The increases in COVID-19 vaccine hesitancy from 2021 to 2022 may have contributed to the low uptake of the updated booster vaccines among adults in the U.S., which was only 20.5% in May 2023 [41]. Given the recent rise in COVID-19 cases and hospitalizations in late 2023 and early 2024 [42], and the new COVID-19 vaccines which were recommended in September 2022 to protect against the latest virus strains [37], being up-to-date with all COVID-19 vaccines is important for protection from severe negative health outcomes of COVID-19 disease. This analysis was among the first to examine changes in hesitancy for vaccines in general and COVID-19 vaccines during the pandemic, and changes in thoughts/attitudes concerning vaccination by hesitancy and vaccination status. This is important for identifying sub-populations that may be at risk for developing gaps in vaccination coverage, and potentially leaving room for disease outbreaks. Given the new COVID-19 vaccines that provides additional protection against new variants, and efforts to catch up on any missed, delayed, or skipped routinely recommended vaccines during the pandemic, this study highlights the opportunity to simultaneously increase vaccine confidence and uptake for all recommended vaccines. Using evidence-based practices such as motivational interviewing and provider recommendations and working with trusted messengers, including religious and community-based organizations, may help increase vaccine confidence and uptake of all recommended vaccines [43,44].

## Data Availability

The data that support the findings of this study are openly available at: https://www.cdc.gov/nchs/rands/data.htm
